# Giant cell tumor of bone: Multimodal approach

**DOI:** 10.4103/0019-5413.32041

**Published:** 2007

**Authors:** AK Gupta, R Nath, MP Mishra

**Affiliations:** Department of Orthopedics, G.S.V.M. Medical College, Kanpur University, Kanpur - 208 002, India; * Department of Pathology, G.S.V.M. Medical College, Kanpur University, Kanpur - 208 002, India

**Keywords:** Giant cell tumor, curettage and bone grafting, wide excision, histological grading of GCT

## Abstract

**Background:**

The clinical behavior and treatment of giant cell tumor of bone is still perplexing. The aim of this study is to clarify the clinico-pathological correlation of tumor and its relevance in treatment and prognosis.

**Materials and Methods:**

Ninety -three cases of giant cell tumor were treated during 1980-1990 by different methods. The age of the patients varied from 18-58 yrs with male and female ratio as 5:4. The upper end of the tibia was most commonly involved (n=31), followed by the lower end of the femur(n=21), distal end of radius(n=14), upper end of fibula (n=9), proximal end of femur(n=5), upper end of the humerus(n=3), iliac bone(n=2), phalanx (n=2) and spine(n=1). The tumors were also encountered on uncommon sites like metacarpals (n=4) and metatarsal(n=1). Fifty four cases were treated by curettage and bone grafting. Wide excision and reconstruction was performed in twenty two cases. Nine cases were treated by wide excision while primary amputation was performed in four cases. One case required only curettage. Three inaccessible lesions of ilium and spine were treated by radiotherapy.

**Results:**

19 of 54 treated by curettage and bone grafting showed a recurrence. The repeat curettage and bone grafting was performed in 18 cases while amputation was done in one. One each out of the cases treated by wide excision and reconstruction and wide excision alone recurred. In this study we observed that though curettage and bone grafting is still the most commonly adopted treatment, wide excision of tumor with reconstruction has shown lesser recurrence.

**Conclusion:**

For radiologically well-contained and histologically typical tumor, curettage and autogenous bone grafting is the treatment of choice. The typical tumors with radiologically deficient cortex, clinically aggressive tumors and tumors with histological Grade III should be treated by wide excision and reconstruction.

Cooper^1^ in 1818 described charaterstics of a tumor which was later on named as giant cell tumour. The term “Giant cell tumor” was coined by Bloodgood.^2^ Jaffe *et al*^3^ differentiated this tumor from other skeletal lesions consisting giant cells and histological grading was done. But it evoked controversy because of untenable clinical correlation. This tumor was also called “Osteoclastoma” by Schajowicz.^4^ The giant cells found in this tumor were differentiated pathogenetically from normal osteoclasts by Lichtenstein.^5^

Since then many large series have been published.^6^–^10^ The histopathological grading has, however been questioned consistently over the years.^8^,^11^,^12^ The ideal treatment still defies this problem. We are presenting an analysis of 93 consecutive cases of giant cell tumor of bone treated between 1980 and 1990 in a general hospital.

## MATERIALS AND METHODS

One hundred and nineteen consecutive cases of histologically proved giant cell tumor (GCT) of bone were treated by us during 1980-1990. The clinical behavior, radiographic and histological features were carefully considered before including a case in this series. The diagnosis was based on the criteria laid down by Jaffe *et al*.^3^ Twenty-six cases which could not be followed adequately for a minimum of three years were excluded from analysis. If a case was lost to follow-up before it completed three years of follow-up, it was not included in this series. Thus the present study includes 93 cases of giant cell tumor.

Fine needle aspiration cytology and /or needle biopsy was done in all cases preoperatively to ascertain diagnosis. The detailed histopathological examination of curetted or excised material was done to exclude any doubtful case.

**Figure 1:**
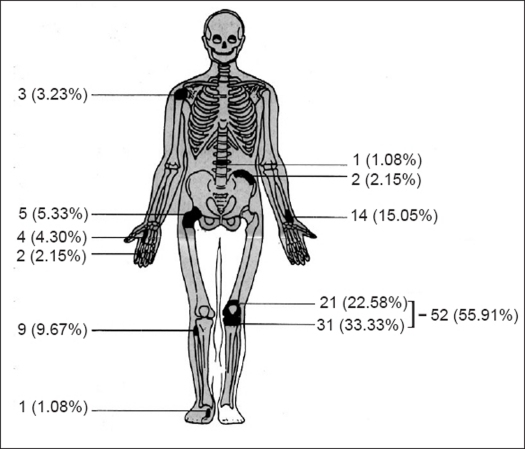
Regional distribution of giant cell tumor of bone found in this series

The age of patients varied from 18 to 58 years. The male to female ratio was 5:4. The lesions were most commonly found [Figure 1] around the bones of the knee joint, (n=52, 55.91%).

The commonest presenting symptom was swelling associated with pain. Pain was aggravated by activity and relieved by rest. When destruction progressed then pain became constant. The sequence of events was pain, swelling and pathological fracture. Duration of symptoms varied from three months to one year. Pathological fracture with involvement of joint was seen in one case (1.07%). However, radiological features of pathological fracture (breach of cortex) were present in 31 cases (33.33%).

Radiologically most of the lesions were found as osteolytic, eccentric and epiphysio-metaphyseal growths. Tumors were present in skeletally mature patients. The cortex was expanded, thinned and perforated or even fractured in 31 cases (33.33%). Articular cartilage was also destroyed in 10 cases, out of this in one case the tumor was invading the joint along with pathological fracture. There was no or minimal periosteal reaction. Typical soap bubble appearance was present in 24 (25.8%) cases. Metastasis to lungs was found in one case. This was a case of malignant giant cell tumor of iliac bone and it appeared three months after radiotherapy though primary tumor regressed in size.

The longitudinal section of excised tumors showed that tumor was well contained even in cases where the cortical shell was deficient except in one case where tumor perforated the articular cartilage and invaded the joint. The tumor consisted mainly of soft grayish pink, friable hemorrhagic tissue. A “capsule” composed of periosteum and fibrous tissue could be made out in most cases. In our cases spontaneous fungation was not observed. Cystic areas of varying size containing thin hemorrhagic fluid and soft yellowish areas of degeneration were observed.

The tumors were classified into three histological grades according to the Campanacci *et al*.^6^ Typical [Grade I] have loosely packed Stroma with no atypism, few mitotic figures and no hyperchromatism. Uniformly distributed, numerous giant cells having multiple nuclei are seen. Aggressive [Grade II] have compact stroma with atypism, frequent mitotic figures and hyperchromatism. The giant cells are less in number unevenly distributed with lesser nucle.

Malignant [Grade III] are frankly sarcomatous with very compact stroma, marked mitosis, marked hyperchromatism. The giant cells are occasional with few nuclei.

In our series 54 cases were graded typical, 28 cases as aggressive and 11 cases as malignant tumor. Eleven tumors were graded primary malignant GCT on initial histopathology. They were iliac bone (n=2), fibula (n=3) lumbar vertebra (n=1) upper end of tibia (n=2), metacarpal (n=1), upper end humerus (n=1) and lower end of femur (n=1). We did not notice any secondary malignant degeneration.^25^,^26^ These tumors differ from anaplastic malignant tumors in respect of better prognosis, less recurrence, amenable to wide excision and better survival rate. We analyzed all cases that recurred. The repeat histopathology was done and compared with initial histopathology but no perceptible significant change was found.

The follow-up was between a minimum of three years to 20 years. Preoperative and postoperative radiographs of all patients were examined. The site and size of the lesion was noted in subsequent follow-up. The local recurrence and pathological fracture were noted. In cases where recurrence was suspected, skiagram of chest was taken as a follow-up protocol. Patient showing clinical evidence of recurrence or increase in the clinical or radiological size of lesion were labeled as recurrence.

## RESULTS

The modality of treatment adopted in different patients is shown in Table 1.

**Table 1:** Primary modalities of treatment and recurrence

Modality of treatment	No. of cases	Percentage	Recurrence (%)
Curettage and autogenous bone grafting	54	58.06	19 (35.18)
Wide excision and reconstruction	22	23.65	1 (4.54)
Wide excision	9	9.67	1 (11.11)
Amputation	4	4.31	-
Curettage	1	1.08	-
Radiotherapy	3	3.23	-
Total	93	100%	-

1. Curettage and autogenous bone grafting (n=54): This treatment was adopted in well-contained typical tumors of weight-bearing bones where radiologically the cortex was not deficient [Figure 2]. It was the most commonly (58.06%) adopted treatment method. However, 19 cases (35.18%) recurred in this group.

**Figure 2:**
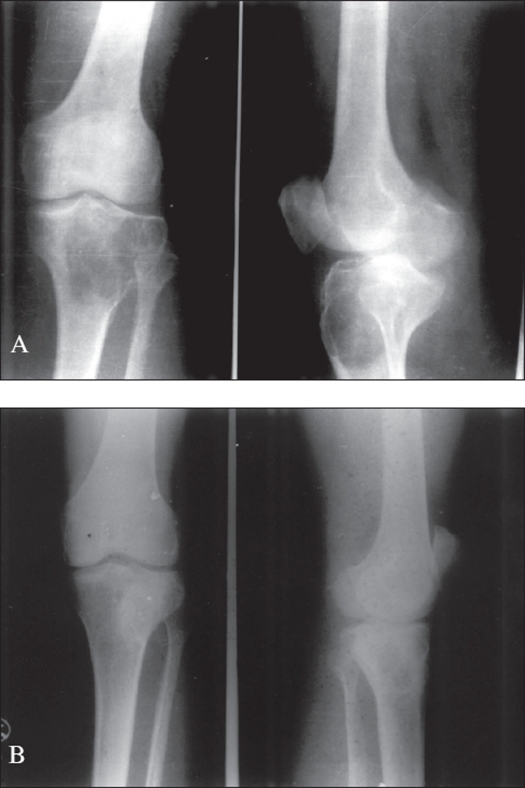
A) X-ray (A.P. and lateral) showing giant cell tumor of the upper end of the tibia. It was graded “typical” on histology. B) Postoperative x-ray four years after curettage and autogenous cancellous bone grafting shows healed lesion with no recurrence.
2. Wide excision and reconstruction (n=22): This procedure was done in 22 cases. In 14 cases of tumor involving the lower end of radius, en bloc resection was done and reconstruction was done by replacing it with ipsilateral upper end of fibula with arthrodesis of wrist [Figure 3]. Fibular graft was fixed by dynamic compression plate (n=5) or Kirschner wire (n=9). There was one recurrence in the lower end of radius, which responded to re-curettage. In this case, three months after the procedure, a soft swelling appeared on the dorsal aspect of the wrist, which was curetted out and sent for histopathology and turned out to be giant cell tumor.

**Figure 3:**
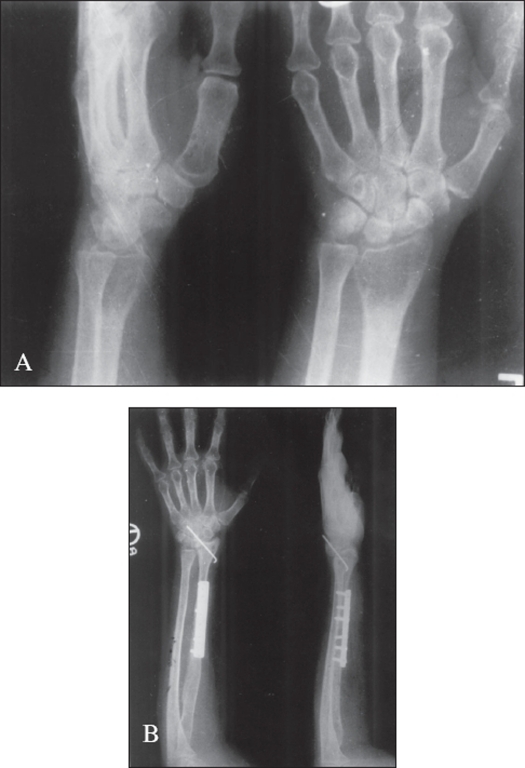
A) Preoperative x-ray (A.P. and lateral) of wrist shows giant cell tumor [Grade: Aggressive] of the lower end of the radius. B) X-ray (AP and lateral) three years after wide excision and autogenous fibular grafting and reconstruction of wrist joint shows no recurrence
3. In GCT involving the upper end of the humerus (n=3) the en bloc resection was done with shoulder arthrodesis by replacing it with autogenous fibular graft. In one case of GCT of the lower end of the femur turnoplasty was done. In one case involving first metatarsal, excision of first metatarsal was done and replaced by free fibular graft and arthrodesis with proximal phalanx and held with kirschnar wire. Three cases of metacarpal involvement were operated for excision and grafting by autogenous iliac bone graft and fixation by Kirschner wire.
4. Wide excision (n=9): The GCTs of bone involving the proximal end of the fibula were treated by excision of the proximal end of fibula. In this group, one case recurred and above knee amputation was done.
5. Amputation (n= 4): In one case, tumor involving the upper end of the tibia had malignant growth (sarcomatous) treated by primary ablation because no other treatment seemed practicable. On five years follow-up there was no chest metastasis or recurrence. In two cases of proximal phalanx amputation was done. One tumor of metacarpal was treated by ray amputation.
6. Curettage alone (n = 1): The small lytic lesion of upper end of tibia was curetted and sent for biopsy. The size of the tumor was less than 1cm. It turned out to be GCT Grade I on histopathology. No autogenous bone grafting was done and lesion healed. At six years follow-up, there was no recurrence.
7. Radiotherapy (n=3): Irradiation was given in the form of cobalt therapy. Two cases were of ilium and one of lumbar verterbra. Ilium cases were having large size tumor with soft tissue infiltration, hence complete excision of the tumor could not be done. Patients were subjected to radiation. Initially there was regression in the left over tumor but ultimately size increased and both patients expired within two years of diagnosis. The case of first lumbar vertebra presented with severe backache and paraparesis. Patient was operated for decompression and operative biopsy. Patient was subjected to radiotherapy. Lesion healed well after radiotherapy but there was no neurological recovery.

The major complications were infection (n=6, 6.46%), transient peroneal nerve palsy (n=6, 6.46%), local recurrence (n=21, 22.58%), pathological fracture (n=1, 1.08%), failed arthrodesis (n=1, 1.08%) and pulmonary metastasis (n=1, 1.08%). Infection healed with antibiotics and transient peroneal nerve palsy recovered in all cases within eight weeks.

Secondary procedure were needed in 21 cases. One case after five years of curettage and bone grafting, presented as recurrence with pathological fracture of lower end of femur. Chest skiagram showed pulmonary metastasis involving both lungs. Mid-thigh amputation was done. Patient expired four months after amputation. In 18 cases of recurrence, repeat curettage and bone grafting was done. There was further recurrence in five cases where amputation was done. One case of GCT of the upper end of the fibula, where initially excision was done, recurrence was noted hence above knee amputation was done. All these tumors were graded typical on initial examination and on repeat histopathology grade of tumor had not changed. On close review of radiographs, it was found that all these patients had deficient cortex. In one case there was failure of arthrodesis of wrist hence bone grafting was done.

## DISCUSSION

Giant tumors are locally aggressive and some may be malignant.^3^,^13^ The benign form of GCT has the intriguing feature of being able, in rare instances, to metastasize despite otherwise benign characteristics.^4^,^14^ The malignant variety of GCT has been defined as a sarcomatous growth that is either primarily juxtaposed to a typical benign focus or occurs after a prolonged interval at the site of a previously treated and documented focus.^6^,^8^,^15^

The concept of staging of musculo-skeletal sarcoma is being debated at present. The TNM system of classification is not applicable to GCT because anatomically GCTs remain intracompartmental for a long time within the well-formed capsule of the periosteum and fibrous tissue.^16^ A histological grading of GCT was first devised by Jaffe *et al*.^3^ They intended to relate the histological features with the clinical course of the tumor, to predict the outcome on that basis. Their grading has subsequently proved to be unreliable.^4^,^11^,^13^

Despite some overlap in histological appearances a majority of GCTs fell into three divisible groups viz. typical, aggressive and malignant. Many observers currently believe that histology alone is a poor index to prognosticate and to predict clinical behavior of tumor.^8^,^11^,^17^ The present study concludes that despite some exceptions the typical tumors having deficient cortex and tumors graded as aggressive or malignant show a distinctly higher recurrence after curettage and bone grafting

By the time a patient seeks institutional treatment in India and other developing countries, the histological life history of a tumor is fully evolved. The histology of such tumors represents the true nature of the tumor and offers a reliable prediction regarding its future behavior. The typical GCT may grow to an enormous size without changing its character and during this process it may undergo cystic degeneration. None of the tumors in our series showed spontaneous ulceration or fungation which occurred only after repeated trauma. Fungation of tumor is not indicative of malignancy but requires wide excision for treatment.

Even clinically and radiographically, GCTs have a wide spectrum. Some lesions grow very slowly and are rarely seen to undergo necrosis, scarring. Others, on the contrary are rapidly aggressive. The tumor may reach the joint surface, enter the joint space and invade the contiguous bone. This can occur in many ways. So in addition to the histological criteria, radiological appearance has an important role in the prognosis of a case. It was seen in our series that if the cortex was deficient radiologically, then curettage and autogenous bone grafting had higher recurrence. It may be because soft tissue infiltration has already taken place at the time of presentation.

Thorough curettage through a sufficiently large window followed by good filling up of a cavity with cancellous bone grafts seems to be justified only in histologically typical tumors that are well contained within an intact cortex. For histologically aggressive tumors the only reliable technique appears to be enbloc resection with conservation of extremity.^18^ The main reasons for poor results of curettage and bone grafting in extensive lesions were tumor recurrence and joint surface collapse. Thus the functional outcomes were even worse than those of patients initially treated with wide resection and reconstructions.^10^

Even after thorough curettage, some microscopic remnants of the tumor cells are bound to be left behind within the walls of the tumor. It is presumed that these remnants are biologically contained by the local immunological response of the reparative cells. Local biological immune response fails in aggressive and malignant tumors. The failure of local immune response explained recurrence in typical GCTs treated by curettage and bone grafting.^16^

Enbloc resection^8^,^13^ is a very reliable method of treating typical as well as aggressive tumors with preservation of the extremity and minimizing the chance of recurrence, however, it has inherent problems. Mittal^19^ reported that in their series enbloc resection yielded best results. We agree with Aithal and Bhaskaranand^20^ that GCT of the lower end of radius are best treated with excision of distal radius and reconstruction by nonvascularised fibula. En bloc resection and reconstruction particularly for femur and tibia requires prolonged protection [one to three years] and involves high rate of graft failure,^9^,^18^ infection and neurovascular complication. In our series we had six cases of transient peroneal nerve palsy and one case of failed athrodesis. En bloc resection and reconstruction for tibia and femur carries high incidence of graft failure requiring surgery in 80% cases. In a series there was 33% incidence of nonunion and 58% fatigue fractures in large bone grafts employed for reconstruction after en bloc resection of bone tumors.^18^ In our series, knee joint (lower end of femur, upper end of tibia and upper end of fibula) was involved in 61/93 cases; we agree with the Su *et al.*^10^ treatment modality that in the knee joint, the integrity of the subchondral bone is critical to the salvageability of the natural joint. They reported poor results of intralesional curettage because of tumor recurrence and joint surface collapse.

Treatment by radiation was given to three cases where the lesion was inaccessible for oncological sound excision. The poor prognosis of tumors in the axial skeleton was shown by Mnyamneh *et al*.^21^ Radiation therapy has been criticized^5^,^22^ due to the fact that the tumor has a high recurrence rate following radiation and that the risk of sarcomatous change is quite high. Encouraging results have been reported by Cryosurgery^23^ and by acrylic cementation.^12^ We did not use both in any of the cases presented. Wide excision and replacement by massive allograft^24^ is a useful procedure but complications are severe and may necessitate amputation.

## CONCLUSION

To decide treatment modality, we took into consideration the clinical, radiological, histological presentation, size of tumor, velocity of tumor growth, bone involved and status of articular cartilage. For radiologically well-contained and histologically typical tumor, we prefer curettage and autogenous bone grafting but in cases of typical tumors with deficient cortex, aggressive and malignant tumors enbloc resection and/or reconstruction was our choice, because these tumors do not respond well to curettage and bone grafting and ultimately recur. The unpredictable clinical behavior of giant cell tumor and its untenable correlation with histopathology and treatment is still an enigma to us.
